# Tumour inflammation signature and expression of S100A12 and HLA class I improve survival in HPV-negative hypopharyngeal cancer

**DOI:** 10.1038/s41598-020-80226-z

**Published:** 2021-01-19

**Authors:** Michael Mints, David Landin, Anders Näsman, Leila Mirzaie, Ramona Gabriela Ursu, Mark Zupancic, Linda Marklund, Tina Dalianis, Eva Munck-Wikland, Torbjörn Ramqvist

**Affiliations:** 1 Department of Molecular Cell Biology, Weizmann Institute of Science, Rehovot, Israel; 2 Department of Surgical and Perioperative Sciences, Urology and Andrology, Umeå University, Umeå, Sweden; 3 Department of Clinical Science, Intervention and Technology (CLINTEC), Karolinska Institutet, Karolinska University Hospital, Stockholm, Sweden; 4 Department of Otorhinolaryngology, Head and Neck Surgery, Karolinska University Hospital, Stockholm, Sweden; 5 Department of Oncology-Pathology, Karolinska Institutet, Bioclinicum J6:20, Karolinska University Hospital, 171 64 Solna, Sweden; 6 Departement of Clinical Pathology and Cytology, Cancer Center Karolinska, R8:02, Karolinska University Hospital, Stockholm, Sweden; 7 Microbiology Department, University of Medicine sand Pharmacy, Grigore T Popa, Iasi, Romania

**Keywords:** Cancer therapy, Head and neck cancer, Tumour biomarkers, Tumour immunology, Tumour virus infections

## Abstract

Hypopharyngeal squamous cell carcinoma (HPSCC) has a very poor prognosis. Local surgery may increase survival, but is often avoided due to significant post-op co-morbidities. Since prognostic markers are lacking, the aim was to find predictive biomarkers that identify patients whose response to oncological treatment is poor and who may benefit from primary surgery to increase survival. Pretreatment biopsies from 23 HPSCC patients, 3 human papillomavirus (HPV) positive and 20 HPV-negative, were analyzed for expression of 750 mRNAs using the Nanostring nCounter IO360 panel in relation to 3-year survival. Validation was performed through immunohistochemistry (IHC) for HLA class I and S100A12 in 74 HPV-negative HPSCC samples. Clustering identified a subset of HPV-negative HPSCC with favorable prognosis and a gene expression signature overexpressing calgranulins and immune genes, distinct from that of HPV-positive HPSCC. Enrichment analysis showed immune signaling, including the tumor inflammation signature, to be enriched in surviving patients. IHC validation confirmed high S100A12 and HLA class I expression to correlate with survival in HPV-negative HPSCC. This shows that immune activity is strongly related to survival in HPV-negative HPSCC. Enrichment of the tumor inflammation signature indicates a potential benefit of immunotherapy. Low expression of both HLA class I and S100A12 could be used to select patients for local surgery.

## Introduction

Hypopharyngeal squamous cell carcinoma (HPSCC) is a clinical challenge. It shows the poorest outcome of all head and neck cancer sites, partly due to frequently being diagnosed in late stages^1^. The total number of cases/year is around 80,000 worldwide^2^ and the main risk factors are smoking and alcohol, although, a small subset of HPSCC is caused by human papillomavirus (HPV)^3–7^. HPSCC caused by HPV, analogous with oropharyngeal squamous cell carcinoma, seems to have a much better clinical outcome than HPV-negative HPSCC^3,4^.

Patients with early stage as well as locoregionally advanced but curable HPSCC are treated with radiotherapy (RT), chemoradiotherapy (CRT), local surgery or combinations of these^1^. Additionally, there are several current clinical trials evaluating immunotherapy with immune checkpoint inhibitors as an optional therapy in HPSCC, however the majority of those are in the recurrent disease/metastatic setting. Several studies, including a recent meta-analysis, have demonstrated better survival for patients undergoing surgery together with RT, as compared to RT/CRT alone, but larger randomised control trials are needed to conclude this^1,8^. Hypopharyngeal surgery is demanding for the patients, who often are in a poor condition, and surgery frequently results in severe side-effects. Since HPSCC often is diagnosed in advanced stages, where surgery, when performed, is extensive, there has been a shift towards oncological treatment during the last decades^1,8^. At the Karolinska University Hospital, oncological treatment has dominated HPSCC treatment since the year 2000. However, if survival could increase with more patients recommended for surgery, there is a need to assess/identify which patients would benefit and there is a need for markers or gene signatures identifying tumors who do not respond satisfactory to oncological treatment alone. Predictive and prognostic biomarkers are thus needed to optimize treatment for patients with limited as well as locoregionally advanced tumors, but also in the metastatic setting where the objective treatment response rate is approximately 20%, it is important to avoid unnecessary toxicity in patients who will not respond.

The aim of this investigation was therefore to find prognostic/predictive biomarkers enabling identification of patients with poor prognosis after oncological treatment. For this purpose, mRNA expression in HPSCC biopsies was evaluated in relation to patient survival using the PanCancer IO 360 panel. This was followed by gene set enrichment analysis, including studying the expression of an 18-gene signature, earlier identified as related to response to PD-L1 inhibitors for several cancer types^9–13^. Furthermore, the expression of two putative biomarkers identified during the initial analysis, S100A12 and HLA class I, was validated to correlate with survival through immunohistochemistry (IHC).

## Results

### Gene expression analysis on the PanCancer IO 360 panel

The expression levels of 750 mRNA transcripts in 23 microdissected HPSCC samples were successfully quantified on the PanCancer IO 360 panel. The included tumors, 3 HPV-positive and 20 HPV-negative, were selected based on completed curative treatment and small tumor size. 10 of the HPV-negative samples were from patients having 3-year relapse-free survival and 10 from patients having a relapse or death by HPSCC within 3 years. To identify gene expression related to patient survival when only receiving radiotherapy or chemoradiotherapy, tumors from patients receiving local surgery were excluded, as were patients who died of causes other than HPSCC within 3 years (Table 1).

**Table 1:** Patient and tumor characteristics.

	Nanostring						Immunohistochemistry	
	All Patients/tumors		HPV negative − NED Patients/tumors		HPV negative − DOD Patients/tumors		Included in survival analysis Patients/tumors	
	n	%	n	%	n	%	n	%
**Total number**	23		10		10		74	
**Gender**								
Male	16	69.6	5	50.0	8	80.0	54	73.0
Female	7	30.4	5	50.0	2	20.0	20	27.0
**Age**								
Mean age	63.9		60.7		68.7		66.6	
**ICD10-code**								
C129	14	60.9	8	80.0	5	50.0	38	51.4
C130	1	4.3	1	10.0	0	0.0	1	1.4
C131	1	4.3	0	0.0	0	0.0	0	0.0
C132	5	21.7	1	10.0	4	40.0	12	16.2
C138	1	4.3	0	0.0	1	10.0	22	29.7
C139	1	4.3	0	0.0	0	0.0	1	1.4
**TNM classification**								
T1	3	13.0	2	20.0	1	10.0	6	8.1
T2	20	87.0	8	80.0	9	90.0	29	39.2
T3	0	0.0	0	0.0	0	0.0	27	36.5
T4/a/b	0	0.0	0	0.0	0	0.0	12	16.2
N0	8	34.8	2	20.0	5	50.0	23	31.1
N1	4	17.4	2	20.0	2	20.0	15	20.3
N2/a/b/c	10	43.5	5	50.0	3	30.0	32	43.2
N3	0	0.0	1	10.0	0	0.0	4	5.4
M0	23	100.0	10	100.0	10	100.0	70	94.6
M1	0	0.0	0	0.0	0	0.0	0	0.0
Mx	0	0.0	0	0.0	0	0.0	4	5.4
**Stage**								
1	1	4.3	0	0.0	1	10.0	2	2.7
2	7	30.4	2	20.0	4	40.0	13	17.6
3	4	17.4	2	20.0	2	20.0	14	18.9
4a/b/c	11	47.8	6	60.0	3	30.0	41	55.4
stage unknown^1^	0	0.0	0	0.0	0	0.0	4	5.4
**HPV/p16-status**^**2**^								
Positive	3	13.0	0	0.0	0	0.0	0	0.0
Negative	20	87.0	10	100.0	10	100.0	74	100.0
**Treatment**								
RT	13	56.5	4	40.0	8	80.0	47	63.5
CRT	1	4.3	1	10.0	0	0.0	7	9.5
(C)RT + regional surgery	8	34.8	5	50.0	2	20.0	20	27.0
Primary local surgery + postoperative RT	1	4.3	0	0.0	0	0.0	0	0.0
Local surgery	0	0.0	0	0.0	0	0.0	0	0.0

*RT* radiotherapy, *CRT* chemoradiotherapy.

^1^Stage unknown due to lack of information on N- and/or M-stage.

^2^Positive status defined by both presence of HPV DNA and overexpression of p16.

### Hierarchical clustering separates samples by prognosis

**Figure 1:**
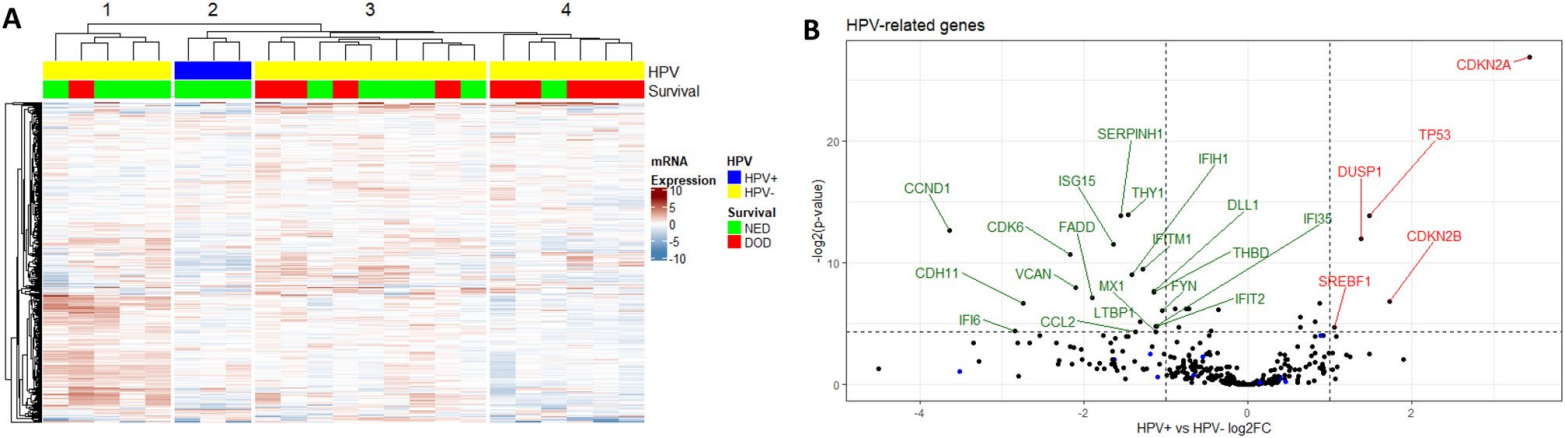
(**A**) Heatmap of all samples. Genes are rows and samples columns. (NED = No evidence of disease, DOD = dead of disease). (**B**) Volcano plot of transcripts differing between HPV-positive and HPV-negative samples. Transcripts higher in HPV-positive samples are marked red. The horizontal line indicates adjusted *p* value of 0.05. Blue dots represent genes in the tumor inflammation signature. Analyses of mRNA expression were performed in R 3.6.2. Heatmaps were plotted with the R package ComplexHeatmap^40^. (https://www.bioconductor.org/packages/release/bioc/html/ComplexHeatmap.html).

Upon initial hierarchical clustering, samples could be divided into four clusters; HPV-positive samples formed one cluster, while HPV-negative samples divided into three groups with high, intermediate and low proportions of survivors, respectively (Fig. 1A).

### Differences between HPV-positive and negative HPSCC are related to the cell cycle

Comparison of the gene expression between HPV-positive and HPV-negative samples presented a  > tenfold overexpression of CDKNA (P16INK4A) in HPV-positive samples, confirming the p16 protein expression as evaluated by IHC. In addition, p53 and p21 showed a strong overexpression while CDK6, CDH11 and CCND1 were among the transcripts down-regulated in HPV positive samples (Fig. 1B, Table 2, Supplementary Table S1).

**Table 2:** Transcripts with > twofold and significant difference in expression between HPV positive and negative HPSCC.

Transcript	Ratio HPV positive/negative HPSCC*	*p* value^†^
**Higher in HPV positive HPSCC**		
CDKN2A	10.81	7.88E−09
CDKN2B	3.31	8.52E−03
TP53	2.80	6.57E−05
DUSP1	2.60	2.46E−04
SREBF1	2.07	3.72E−02
**Lower in HPV positive HPSCC**		
FYN	0.48	1.47E−02
IFIT2	0.46	3.46E−02
MX1	0.46	4.89E−02
IFI35	0.46	3.46E−02
DLL1	0.45	4.71E−03
THBD	0.45	5.05E−03
IFITM1	0.41	1.38E−03
LTBP1	0.40	2.70E−02
CCL2	0.39	4.89E−02
IFIH1	0.38	1.84E−03
THY1	0.36	6.36E−05
SERPINH1	0.34	6.57E−05
ISG15	0.32	3.26E−04
FADD	0.27	6.79E−03
VCAN	0.23	3.92E−03
CDK6	0.22	5.77E−04
CDH11	0.15	9.58E−03
IFI6	0.14	4.69E−02
CCND1	0.08	1.55E−04

*2-log ratios transformed into linear ratios.

^†^Adjusted for false discovery rate.

### Immune genes linked to survival in HPV-negative HPSCC

**Figure 2:**
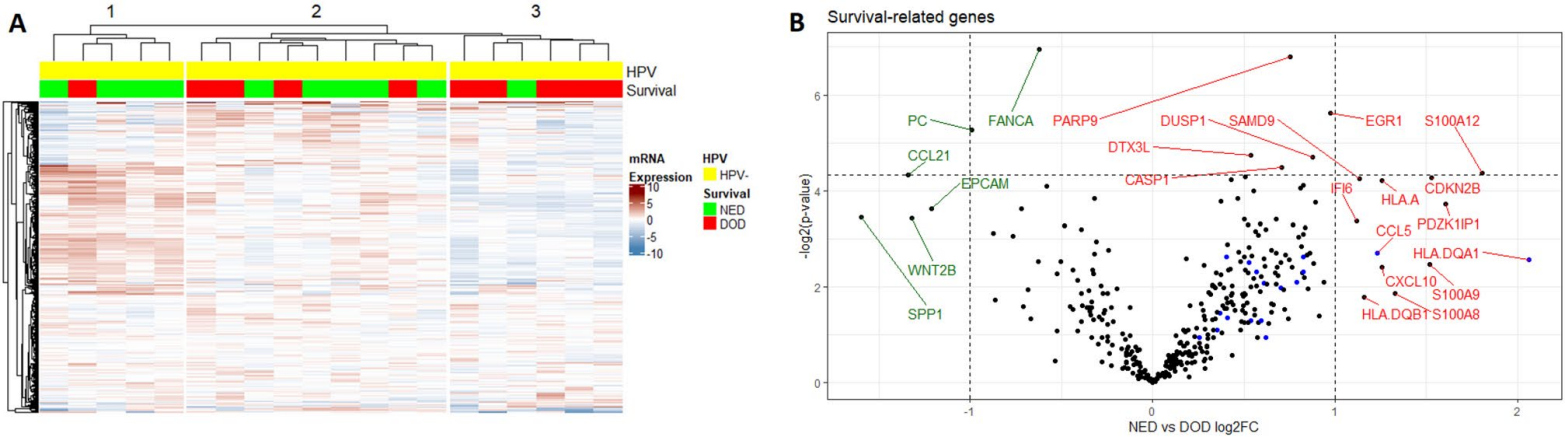
(**A**) Heatmap of all HPV-negative samples. Genes are rows and samples columns. (**B**) Volcano plot of transcripts differing between surviving and non-surviving patients with HPV-negative HPSCC. Transcripts higher in survivors are marked red. Unadjusted *p* values are presented and the horizontal line indicates an unadjusted *p* value of 0.05. Blue dots represent genes in the tumor inflammation signature. Analyses of mRNA expression were performed in R 3.6.2. Heatmaps were plotted with the R package ComplexHeatmap^40^. (https://www.bioconductor.org/packages/release/bioc/html/ComplexHeatmap.html).

When HPV-negative samples were evaluated separately, they clustered into the same three groups described above. Although surviving patients were clearly enriched in cluster 1, and underrepresented in cluster 3, this difference was not significant (*p* = 0.07, Fig. 2A). After adjusting for multiple testing, no transcripts remained significantly different between the patients who responded to treatment (no evidence of disease, NED) and those who did not (dead of disease, DOD) (Fig. 2B, Table 3, Supplementary Table S1). Genes found to have large differences in expression between the NED and the DOD group, as well as those with significant unadjusted *p* values were used to construct heatmaps shown in Supplementary Fig. S1. Genes with higher expression in the NED group included several calgranulins (S100A8, A9 and A12) showing similar expression patterns, and HLA class I and II genes (HLA-A, HLA-DQA1 and HLA-DQB1), while those with lower expression in the NED group included SPP1, PC and FANCA.

**Table 3:** Transcripts with > twofold and/or significant† differences in expression between the NED and DOD group in HPV negative HPSCC.

Transcript	Ratio NED/DOD*	*p* value^†^
**Higher in NED**		
HLA-DQA1	4.17	1.69E−01
**S100A12**	3.50	4.84E−02
PDZK1IP1	3.04	7.58E−02
CDKN2B	2.89	5.21E−02
S100A9	2.87	1.81E−01
S100A8	2.51	2.74E−01
**HLA-A**	2.39	5.37E−02
CXCL10	2.39	1.87E−01
CCL5	2.35	1.54E−01
HLA.DQB1	2.23	2.89E−01
SAMD9	2.19	5.24E−02
IFI6	2.17	9.66E−02
EGR1	1.96	2.02E−02
DUSP1	1.84	3.85E−02
PARP9	1.69	9.01E−03
CASP1	1.63	4.44E−02
DTX3L	1.45	3.76E−02
**Lower in NED**		
**FANCA**	0.65	8.08E−03
PC	0.50	2.60E−02
EPCAM	0.43	8.16E−02
WNT2B	0.40	9.32E−02
CCL21	0.40	4.98E−02
SPP1	0.33	9.19E−02

*2-log ratios transformed into linear ratios.

^†^Not adjusted for false discovery rate.

When comparing clusters 1 and 3 against each other, a large number of genes were found to be differentially expressed (Supplementary Fig. S2, Supplementary Tables S1 and S2). The majority of transcripts up-regulated in cluster 1, consisting of patients with good prognosis, were immune-related and included IDO1, NKG7, CD27, CCL5, CXCL9 and CXCL10.

### Immune signaling pathways are enriched in HPV-negative patients with favorable prognosis

Gene set enrichment analysis (GSEA) was performed to identify cell signaling pathways correlating with clinical parameters (Supplementary Fig. S3, Supplementary Table S3). No pathways were significantly enriched in HPV-positive samples after adjusting for multiple testing. However, without adjusting the *p* values, cell cycle checkpoints were enriched in HPV-positive samples, while cytokine and extracellular matrix signaling were comparatively enriched in HPV-negative samples. When comparing the NED and DOD groups, as well as cluster 1 vs. cluster 3, immune and interferon signaling were significantly enriched for the NED group and cluster 1, with both groups containing tumors responding to treatment. Tyrosine kinase signaling was enriched among non-responders.

### Gene expression in relation to tumor inflammation signature for HPV negative HPSCC

**Figure 3:**
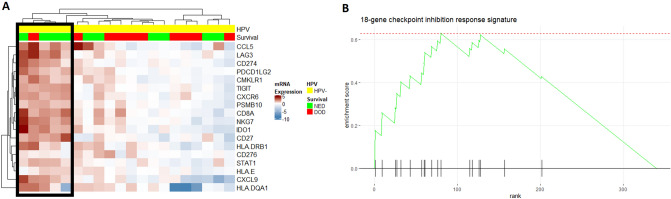
(**A**) Heatmap of all HPV-negative samples for the 18 genes in the tumor inflammation signature. The black box marks samples from cluster 1 in (**B**) Gene set enrichment plot for the 18-gene signature in relation to survival. Genes in the signature are marked on the x-axis and ranked from left to right based on their log2-fold change between tumors responding to treatment ant non-responding. Analyses of mRNA expression were performed in R 3.6.2. Heatmaps were plotted with the R package ComplexHeatmap^40^. (https://www.bioconductor.org/packages/release/bioc/html/ComplexHeatmap.html).

An 18-gene signature (CCL5, CD27, CD274, CD276, CD8A, CMKLR1, CXCL9, CXCR6, HLA-DQA1, HLA-DRB1, HLA-E, IDO1, LAG3, NKG7, PDCD1LG2, PSMB10, STAT1, TIGIT) has recently been proposed to be predictive for a clinically favorable response to treatment with PD-L1 inhibitors^9–11^. These genes were all overexpressed in tumors responding to treatment (Fig. 1B), and were markedly enriched in the cluster of HPV-negative patients with favorable prognosis (Supplementary Fig. S2). Confirming these observations, GSEA showed this gene signature to be significantly enriched in tumors responding to treatment (Supplementary Fig. S3, Supplementary Table S3). Clustering all HPV-negative samples based on their expression of these genes showed that samples with high expression of all or nearly all of these genes formed a cluster where all but one patient survived (Fig. 3A). Notably, this cluster of surviving patients consisted of the same five samples as those forming Cluster 1 in Fig. 2A, where 4/5 patients survived. This shows that the genes in the tumor inflammation signature drive clustering even when hundreds of genes are involved. To further illustrate the impact of this gene signature on the survival of patients with HPV-negative HPSCC, Fig. 3B shows a pathway enrichment plot for the 18-gene signature, which was significantly enriched in surviving patients.

### Expression of S100A12 and HLA class I is linked to survival

**Figure 4:**
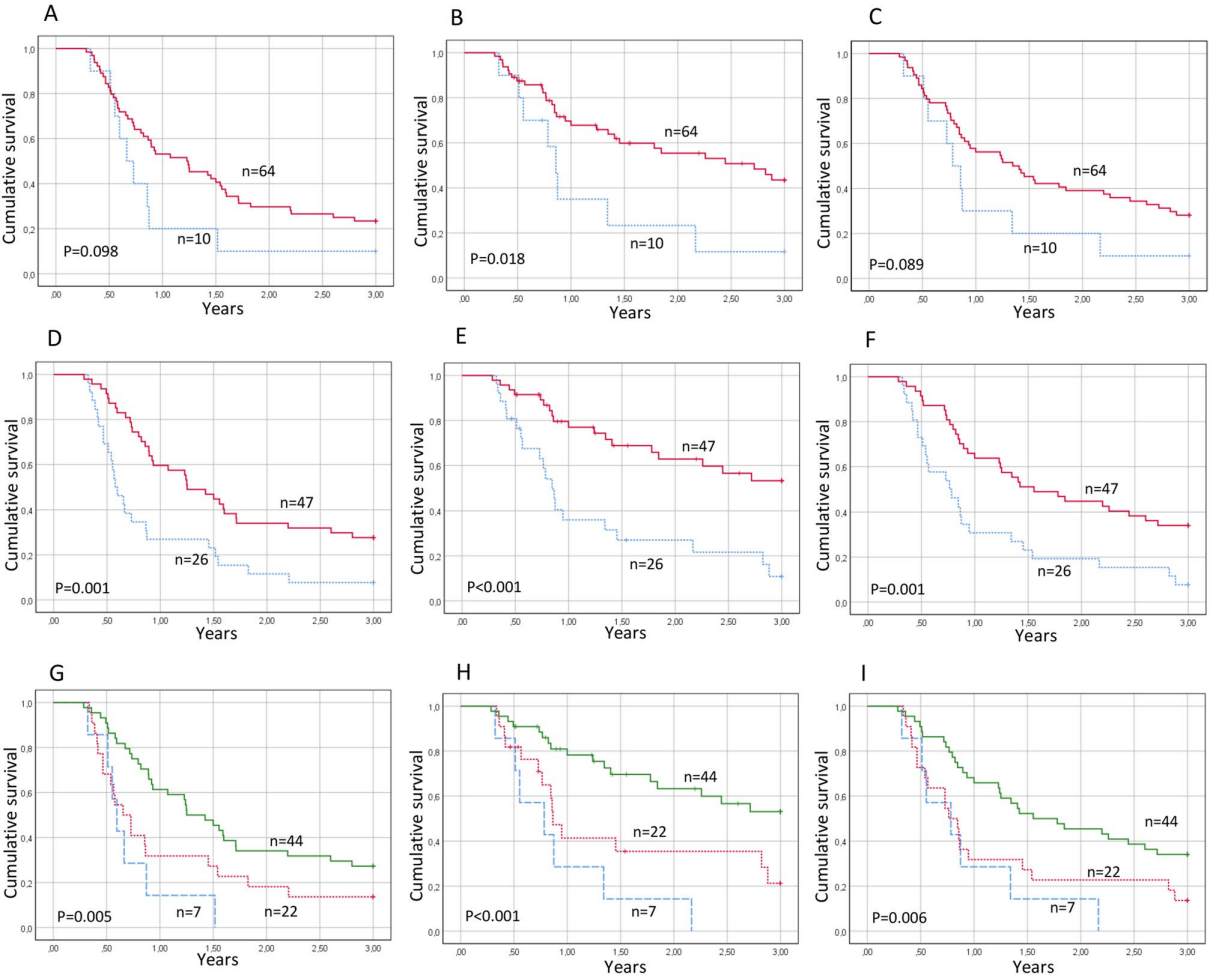
Kaplan–Meier curves presenting progression free (**A**,**D**,**G**), disease free (**B**,**E**,**H**) and overall survival (**C**,**E**,**I**) for patients with HPV-negative HPSCC in relation to S100A12 or HLA class I expression. (**A**–**C**) S100A12 expression dichotomized between fraction of positive cells more (red) or less (blue) than 33%. (D-F) HLA class I expression dichotomized between absent/low (blue) and medium/high (red). (**G**–**I**) Combined expression of S100A12 and HLA class I. Both high (green), one high, one low (red) or both low (blue). Notches denotes censored.

Several S100 genes; S100A8, S100A9 and S100A12, showed an increased expression in HPV-negative tumors responding to treatment (Fig. 2B). Even though none of these genes was significantly overexpressed by itself, the fact that three related proteins all showed highly increased expression warranted further study. To validate this finding in a larger dataset, S100A12, showing the largest difference between responders and non-responders (3.5 times higher in responders) was chosen for further analysis by IHC (Supplementary Fig. S4). Seventy-four tumors from patients who had completed curative treatment (Table 1) were consequently analyzed for their expression of S100A12. A minority of tumors (10/74) showed complete absence or only weak expression in a minor part (< 33%) of the tumor. When progression-free, disease-specific and overall survival (PFS, DSS and OS) were analyzed in relation to the fraction of S100A12-positive cells, DSS was found to be significantly worse in patients with tumors having a low expression of S100A12 (*p* = 0.018) (Fig. 4A–C). For PFS and OS, low expression was also associated with low survival, although not significantly so (*p* = 0.098, *p* = 0.089, resp.).

Since immune signaling pathways were highly enriched, and several HLA antigens (HLA-A, HLA-DQA1 and HLA-DQB1) were overexpressed in patients with favorable prognosis, HLA class I A,B,C expression was also validated by IHC. PFS, DSS and OS were all significantly better for patients with medium or high HLA class I expression (*p* = 0.001, *p* < 0.001 and *p* = 0.001 for PFS, DSS and OS, resp.) (Fig. 4D–F).

Tissue expression levels of S100A12 and HLA class I were also analyzed together and found to be significantly correlated with PFS, DSS and OS (*p* = 0.005, *p* < 0.001 and *p* = 0.006, resp.) (Fig. 4G–I). Notably, none of the 7 patients with tumors lacking expression of both of these proteins survived for 3 years, and all had a PFS of at most 1.5 years.

FANCA, which was overexpressed in non-responders (Table 3) was also analyzed by IHC. However, no significant association between FANCA expression and survival was detected (data not shown).

### Hazards analysis of S100A12 and HLA class I together with clinical parameters in relation to survival

S100A12 and HLA class I expression in relation to PFS, DSS and OS was also evaluated by univariate and multivariate Cox proportional hazards analyses (Table 4). Upon univariate analysis, hazards ratios for having a tumor with low S100A12 expression were 1.82, 2.51 and 1.85 for PFS, DSS and OS respectively, while the corresponding figures for low HLA class I expression were 2.31, 3.32 and 2.34. In the multivariate analysis, S100A12 and HLA class I were analyzed with sex, age, tumor size and nodal status as covariates. For PFS and OS, both HLA class I and age were significant in the multivariate analysis, while for DSS only HLA class I remained significant. When S100A12 and HLA class I expression were included as the only covariates in a multivariate analysis, HLA class I remained statistically significant in relation to PFS, DSS and OS, while S100A12 did not, demonstrating that the expression of these proteins is not quite independent (data not shown).

**Table 4:** Univariate and multivariate Cox regression analysis for 3-year survival in patients with HPV negative HPSCC.

	Univariate			Multivariate^ǁ^		
Factor	HR	95% CI	*p* value	HR	95% CI	*p* value
**3-year progression free survival (PFS)**						
Sex*	0.69	(0.502–0.934)	**0.017**	0.64	(0.327–1.253)	0.193
Age^†^	1.04	(1.014–1.064)	**0.002**	1.03	(1.008–1.058)	**0.01**
Tumor size^‡^	0.55	(0.325–0.932)	**0.026**	0.77	(0.423–1.385)	0.38
Nodal status^§^	0.65	(0.365–1.162)	0.146	0.70	(0.383–1.294)	0.259
HLA class I^§^	2.31	(1.361–3.925)	**0.002**	1.97	(1.119–3.47)	**0.019**
S100A12^?^	1.82	(0.886–3.742)	0.103	1.45	(0.688–3.071)	0.073
**3-year disease specific survival (DSS)**						
Sex*	0.26	(0.100–0.664)	**0.005**	0.35	(0.127–0.938)	0.037
Age^†^	1.03	(0.999–1.061)	0.058	1.02	(0.990–1.051)	0.202
Tumor size^‡^	0.43	(0.218–0.829)	**0.012**	0.80	(0.373–1.696)	0.553
Nodal status^§^	0.074	(0.224–1.072)	0.074	0.61	(0.273–1.375)	0.235
HLA class I^§^	3.32	(1.745–6.331)	** < 0.001**	2.85	(1.418–5.724)	**0.003**
S100A12^?^	2.51	(1.140–5.528)	**0.022**	2.11	(0.922–4.813)	0.077
**3-year overall survival (OS)**						
Sex*	0.65	(0.47–0.91)	**0.012**	0.56	(0.276–1.136)	0.108
Age^†^	1.04	(1.016–1.068)	**0.001**	1.04	(1.009–1.062)	**0.008**
Tumor size^‡^	0.52	(0.30–0.89)	**0.018**	0.71	(0.391–1.307)	0.275
Nodal status^§^	1.45	(0.801–2.637)	0.218	0.81	(0.434–1.495)	0.492
HLA class I^§^	2.34	(1.37–4.01)	**0.002**	1.92	(1.083–3.413)	**0.026**
S100A12^?^	1.85	(0.90–3.81)	0.094	1.55	(0.74–3.23)	0.240

S100A12 expression dichotomized between 1/2 and 3/4.

HLA class I and S100A12 evaluated separately in the multivariate analysis.

*HR* hazard ratio, *CI* confidence interval.

*Sex evaluated female vs male.

^†^Age evaluated as a continuous variable.

^‡^T-stage dichotomized between T1 + T2 vs T3 + T4.

^§^HLA class I expression dichotomized between absent/low and medium/strong.

^ǁ^Including all four factors.

### Combined analysis of HLA class I expression and CD8-positive tumor infiltrating lymphocytes predicts survival

Sixty-eight of the 73 tumors analyzed for HLA class I expression were in an earlier study evaluated for numbers of CD8-positive tumor infiltrating lymphocytes (CD8 + TILs)^14^. When data on CD8 + TILs was combined with HLA class I expression from the present study there was a very strong and significant difference in survival between patients having HPSCC with low HLA class I expression and low number of TILs and those with high HLA class I expression and high number of CD8 + TILs, PFS, DSS and OS (*p* = 0.004, *p* < 0.001 and *p* = 0.005, for PFS, DSS and OS resp., Supplementary Fig. S5).

## Discussion

In the present study, expression of cancer and immune related genes in HPSCC biopsies was analyzed in relation to treatment outcome. A specific aim was to identify prognostic markers to help select HPSCC patients who, because of their poor prognosis, would benefit from surgery despite the side-effects. Gene expression analysis combined with enrichment analysis demonstrated that immune activation is linked to survival in HPV-negative HPSCC. The analysis indicated high expression of several HLA class I and II antigens as well as several calgranulins, S100A8, A9 and A12 as related to better treatment response. Expression of HLA class I (A,B,C) and S100A12 was further validated by IHC as markers of improved clinical outcome.

When performing gene set enrichment analysis (GSEA), immune signaling was significantly enriched in tumors responding to treatment while receptor tyrosine kinase (RTK) signaling was enriched in non-responding tumors. Tumor immunity and CD8 + lymphocyte infiltration are positive prognostic factors in many cancers including HPSCC, while RTK signaling is a negative prognostic factor^14–19^. The immune signatures here were characterized by the antigen presentation system through overexpression of interferons, HLA class I and CD8.

An 18-gene signature for inflammation response, suggested to be predictive for a favorable response to treatment with PD-L1 inhibitors^9–11^, and which was enriched in responding patients upon GSEA, was also studied separately in relation to survival. Clustering using these 18 genes identified 5/20 HPV-negative patients as potential responders to PD-L1 treatment. Notably, 4/5 of these patients survived for at least 3 years, indicating that these tumors respond better to RT/CRT treatment as well.

The correlation between high expression of HLA class I (A, B, C) and survival in HPSCC was not unexpected, as a number of studies have demonstrated similar associations for HPV-negative HNSCC from other subsites^20–25^. Despite the fact that HPSCC in general has a very poor prognosis, patients with tumors showing high HLA class I expression had a much improved treatment outcome, indicating the importance of tumor recognition by the immune system for these tumors. Further highlighting the importance of anti-tumor immunity, CD8 + TIL numbers have been linked to increased patient survival in several studies of HPSCC^14,17,26^. Here, the combined evaluation of HLA class I expression and CD8 + TILs demonstrated a major difference in survival between patients with tumors having high HLA class I expression and high numbers of CD8 + TILs *vs* tumors showing low values for both. This contradicts a previous HPSCC study, where no association was found between HLA class I expression and survival, although HLA class I was found to correlate with CD8. However, this study was limited to late-stage patients receiving neoadjuvant chemotherapy, which could explain the discrepancies in results^17^.

Notably, the HLA class II antigens HLA-DQA1 and HLA-DQB1 were also overexpressed in responders on the mRNA level. A positive correlation between high expression of HLA class II antigens and favorable clinical outcome has earlier been shown by IHC for HPV-negative oropharyngeal squamous cell carcinoma (OPSCC)^22^.

Low expression of S100A8, A9 and A12 (calgranulin A, B and C) mRNA was related to worse prognosis. Validation of S100A12, showing the largest expression difference between responders and nonresponders, showed tumors with absent or very low expression to have significantly worse clinical outcome. S100A8, A9 and A12 belong to a subgroup of S100 proteins, calgranulins, involved in senescence, aging and inflammation response. They are particularly expressed by cells in the innate immune system, e.g. neutrophils and macrophages^27–30^. S100 proteins have also been associated with epithelial differentiation in a single-cell study of head and neck cancer^31^. While S100A8 and A9 can act together by forming heterodimers, calprotectin, S100A12 functions as a homodimer. S100A8/S100A9 is often upregulated in cancer and is involved in inducing a pro-inflammatory response^32^. High S100A8 expression has been associated with increased disease specific and overall survival in both breast cancer and HNSCC^33,34^. In OPSCC and oral cancer, S100A8/S100A9 is often downregulated, associated with lower tumor grading, upregulation of apoptosis-related genes, decreased EGFR expression, epithelial differentiation and decreased migration and invasion^35,36^. While S100A12 is constitutively expressed by neutrophils and inducible in macrophages, expression in epithelial cells can lead to growth arrest^27,37^. S100A12 has received less attention than S100A8 and S100A9, but all three proteins were the focus of a study on OPSCC by Funk et al., where high expression of S100A8 and/or S100A12 were favorable prognostic factors^38^. A difference between the OPSCC study by Funk et al. and the present study was that much fewer HPSCC had low S100A12 expression. This may be similar to the lower levels of S100A8/S100A9 expression in OPSCC and OSCC as compared to other head and neck sites^35^.

There are several limitations caused by the small sample size. Since the number of samples was low and a large set of transcripts was analyzed, no transcript was significantly associated with survival after adjusting for multiple testing. In addition, the sample size led to patients receiving RT and CRT being analyzed together. It is thus possible that genes associated with response to only one of these treatments remained undetected. Another possible confounder is the higher age and lower frequency of CRT in the non-response group, while in the response group the tumors were on average of a higher stage. Thus, there is a need to further validate and extend the results presented in this study in a larger dataset.

In conclusion, we have identified an immune signature as a predictor of survival in HPV-negative HPSCC and validated S100A12 and HLA class I, particularly when combined with CD8, as markers for treatment response. This opens up prospects for applying immunotherapy and checkpoint inhibition in HPSCC, as well as using absent S100A12 and HLA class I expression as a means to indicate patients for whom the survival benefits of surgery probably outweigh the side effects.

## Methods

### Patients and tumor biopsies

Hypopharyngeal cancer biopsies with the ICD-10 codes C12.9 (pyriform sinus), C13.0 (postcricoid region), C13.1 (aryepiglottic fold, hypopharyngeal aspect), C13.2 (posterior wall of hypopharynx), C13.8 (overlapping sites of hypopharynx) and C13.9 (hypopharynx, unspecified location) were derived from two previously analyzed and published patient cohorts, covering in total 191 patients, treated 2000–2013 at the Karolinska University Hospital in Stockholm^3,4^. Pretreatment formalin-fixed paraffin-embedded (FFPE) biopsies from these cohorts were earlier examined for the presence of HPV DNA from 24 or 27 different HPV types by a Luminex bead-based assay and for the expression of p16INK4A (p16) in relation to clinical outcome^3,4^. Only samples both positive for HPV DNA and overexpressing p16 were considered as HPV positive. All three HPV positive samples were positive for HPV type 16. HPV DNA positive/p16 negative samples were not included in the study. Patient and tumor characteristics are presented in Table 1.

The study was performed according to permission 2009/1278-31/4 from the Regional Ethics Committee, Karolinska Institutet. Informed consent was obtained from all patients. All methods were carried out in accordance with relevant guidelines and regulations. All experimental protocols were approved by the Dept. Oncology-Pathology, Karolinska Institutet.

### Selection of samples for gene expression analysis and immunohistochemistry

Inclusion criteria for selection of biopsies for gene expression analysis were complete patient records, small tumor size (T2 or T1), completed curative treatment and where patients either had a 3-year relapse free survival (group NED, no evidence of disease) or had died within 3 years due to the disease (group DOD, dead of disease). In addition, for HPV negative tumors, only patients who did not undergo local surgery were included. For immunohistochemistry, the criteria for inclusion were complete patient records, completed curative treatment and no local surgery.

Ten of the HPV-negative patients had 3 years disease-free survival, while the remaining 10 all died from the disease within 3 years. In the latter group, eight patients relapsed before death due to their cancer, while two had residual disease. All three patients with HPV positive tumors, defined as being both HPV DNA and p16 positive, survived 3 years and were relapse free.

### Sample preparation and nanostring assay

Six 5 μm cuts/FFPE HPSCC biopsies were obtained using a microtome and mounted on PENMembrane 2.0 μm membrane slides (No.11505158) (Leica Microsystems, Wetzlar, Germany). Before microdissection cuts were deparaffinized for 2 × 15 min in fresh xylene, then rehydrated in decreasing percentages of ethanol (5 min each in 100%, 95%, 70%, 50%, 0% ethanol) and stained for 30 s with hematoxylin. Areas with tumors from the six cuts/tumor were then laser microdissected using a Leica LMD 7000 microscope (Leica Microsystems, Wetzlar, Germany) and the Laser Microdissection System (version 7.6.5684), pooled in microcentrifuge tubes containing PKD buffer from the RNeasy FFPE kit (Qiagen, Halden, Germany) and kept on ice.

RNA was extracted directly after microdissection using the RNeasy FFPE kit according to manufacturer's instructions. RNA concentrations were evaluated using the the Qubit RNA HS Assay Kit with the Qubit 4 Fluorometer (Thermo Fisher Scientific, Waltham, MA, USA). All included samples had RNA concentrations > 7 ng/ul and all except one > 18 ng/ul. No amplification step was included before analysis on the nanostring panel.

Gene expression was analyzed at the KIgene facility, Center for Molecular Medicine, Karolinska Institute, using the nCounter IO360 panel, (NanoString Technologies, Seattle, WA, USA) according to manufacturer's instructions, using the nCounter Sample Prep Station with FLEX configuration (NanoString Technologies) and the nCounter Digital Analyzer 5 s (NanoString Technologies) for reading the samples.

### Immunohistochemistry and evaluation of HLA class I, S100A12 and FANCA

Expression of HLA class I was analyzed using the mouse monoclonal HLA class I ABC antibody (ab70328, Abcam, Cambridge, UK), recognizing the heavy chains of human HLA class 1 A, B and C, diluted 1:1000. Staining was performed essentially as described previously with Vectastain Elite ABC HRP kit, PK-6100, and DAB Peroxidase (HRP) Substrate Kit SK-4100, both from Vector Laboratories, Burlingame, CA, USA^24^. As secondary antibody, biotinylated anti-mouse IgG (BA-2000, Vector Laboratories), diluted 1:200, was utilized.

Evaluation of staining was performed by three investigators (AN, TR and RU), blind to clinical outcome. In case of disagreement, a consensus decision was made. Fractions of HLA class I expressing cells were evaluated semi-quantitatively in five grades: 0 (0%), 1 (1–25%), 2 (26–50%), 3 (51–75%), and 4 (76–100%). Intensity of expression was scored on a four-tier scale as absent, weak, medium and strong staining. For analysis of survival, intensity of expression was dichotomized into absent/weak or medium/strong. Examples of staining are presented in Supplementary Fig. S4.

S100A12 expression was analyzed using the Calgranulin C (19F5): sc-101347 mouse monoclonal antibody from Santa Cruz Biotechnology (Dallas, TX, USA), diluted 1:60. Staining was performed as for HLA class I above and evaluated blind by three investigators (AN, TR and RU). Intensity of expression and fraction of positive cells was evaluated in a similar manner as Funk et al.^38^. The fraction of S100A12 positive cells was evaluated as 1 = 0%, 2 = 1–33%, 3 = 34–67% and 4 = 68–100%. Intensity was evaluated as 1 = negative, 2 = weak, 3 = medium and 4 = strong. However, fraction and intensity were not combined into a score as described by Funk et al.^38^. For evaluation of survival, the fraction of positive cells was dichotomized into 1 + 2 (0–33%) or 3 + 4 (34–100%). Examples of staining are presented in Supplementary Figur. S4.

Expression of FANCA was analyzed with the rabbit anti-FANCA polyclonal antibody ASJ-C2020F from Nordic Biosite (Stockholm, Sweden) at a dilution of 1:100. As secondary antibody biotinylated Goat Anti-Rabbit IgG Antibody (BA-1000), diluted 1:200, was utilized. The intensity and frequency of FANCA was evaluated as number of 10-percentiles of cells with absent, weak, intermediary or strong staining. For evaluation of FANCA expression in relation to survival, fractions of cells with intermediary and strong staining were combined.

### Evaluation of patient survival in relation to IHC analysis

For analysis of survival in relation to expression of S100A12 or HLA class I expression by IHC, survival was measured in days from diagnosis until an event occurred, or until three years after diagnosis when patients were censored. Survival was evaluated as in the study by Landin et al.^14^. The Kaplan–Meier estimator was used for evaluation of three-year progression free survival (PFS), disease specific survival (DSS) and overall survival (OS). For PFS, disease recurrence, or death of any cause before three years were considered as events. For evaluation of disease specific survival (DSS), death with documented relapse was considered as an event while patients that had deceased before three years from other causes were censored at time of death. For OS, death of any cause before three years was considered as an event. Differences in the survival of patients in relation to S100A12 or HLA expression were evaluated using the log-rank test. Univariate and multivariate hazard ratios (HR) were calculated using Cox proportional hazards regression analysis. Calculations and analyses were performed using IBM SPSS Statistics, version 26.0.

### Nanostring data analysis

Only endogenous mRNA transcripts were kept for downstream analysis, removing housekeeping genes. In order to increase power upon multiple testing, transcripts were filtered by mean and variance, keeping only those transcripts having above median variance and above median gene expression. This resulted in 332 out of 750 endogenous mRNA transcripts remaining (Supplementary Fig. S6). From the 18-gene tumor inflammation signature of response to PD-1 inhibition (9–13), seven genes that did not pass the mean and/or variance thresholds were nevertheless kept for downstream analysis. All raw nanostring counts are found in Supplementary Table S4. The expression data were log2-transformed and median-centered by sample before analysis, and re-centered whenever sub-group analyses not including all patients or transcripts were performed.

Student’s t-test was used for comparing continuous variables and Fisher’s exact test for comparing categorical variables. *p* values were adjusted for multiple testing using the Benjamini–Hochberg method. *p* values below 0.05 were considered significant.

For gene set enrichment analysis, enrichment scores and significance values were calculated as described in Subramanian et al.^39^. Highly similar pathways were collapsed into one in order to show only independently enriched pathways. All analyses of mRNA expression were performed in R 3.6.2. Heatmaps were plotted with the R package ComplexHeatmap^40^. For hierarchical clustering, Euclidean distance and complete linkage were used as parameters GSEA was performed using the fgsea R package^41^. Functional pathways were taken from Reactome^42^.

## Data availability

The nanostring data generated and analysed during this study are included in Supplementary Table S4. Clinical data used for survival analysis based on evaluation by IHC are not publicly available, due to regulations on transfer of such data. However, the data utilized in the analysis in the present study is available from the corresponding author on reasonable request.

## Supplementary Information

Supplementary Figures.

Supplementary Table 1.

Supplementary Table 2.

Supplementary Table 3.

Supplementary Table 4.
