# Supplementary material for: Tumour inflammation signature and expression of S100A12 and HLA class I improve survival in HPV-negative hypopharyngeal cancer
Source: Sci Rep. 2021 Jan 19;11:1782. doi: 10.1038/s41598-020-80226-z (PMC7815817; doi:10.1038/s41598-020-80226-z)

Supplementary information

Tumour Inflammation Signature and Expression of S100A12 and HLA Class I in HPV-negative Hypopharyngeal Cancer Improve Survival

Michael Mints, David Landin, Anders Näsman, Leila Mirzaie, Ramona Gabriela Ursut, Mark Zupancic, Linda Marklund, Tina Dalianis, Eva Munck-Wikland, Torbjörn Ramqvist

Supplementary Figure S1. Heatmaps of all HPV-negative samples and (A) Genes with more than two-fold expression difference between surviving and non-surviving patients. (B) Genes with unadjusted $p<0.05$ upon comparison between surviving and non-surviving patients. (NED=No evidence of disease, DOD=dead of disease). Analyses of mRNA expression were performed in R 3.6.2. Heatmaps were plotted with the R package ComplexHeatmap. (https://www.bioconductor.org/packages/release/bioc/html/ComplexHeatmap.html)

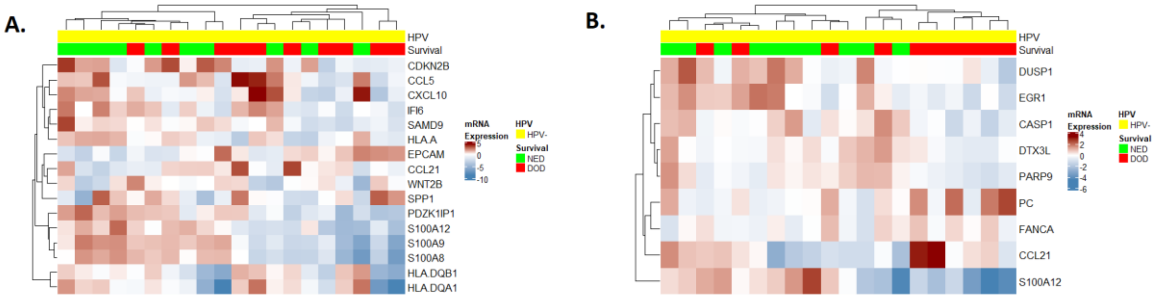

Supplementary Figure S2. Volcano plot of genes differing between clusters 1 and 3. Genes higher in cluster 1 are marked red. The horizontal line indicates adjusted p-value of 0.05. Blue dots represent genes in the tumor inflammation signature.

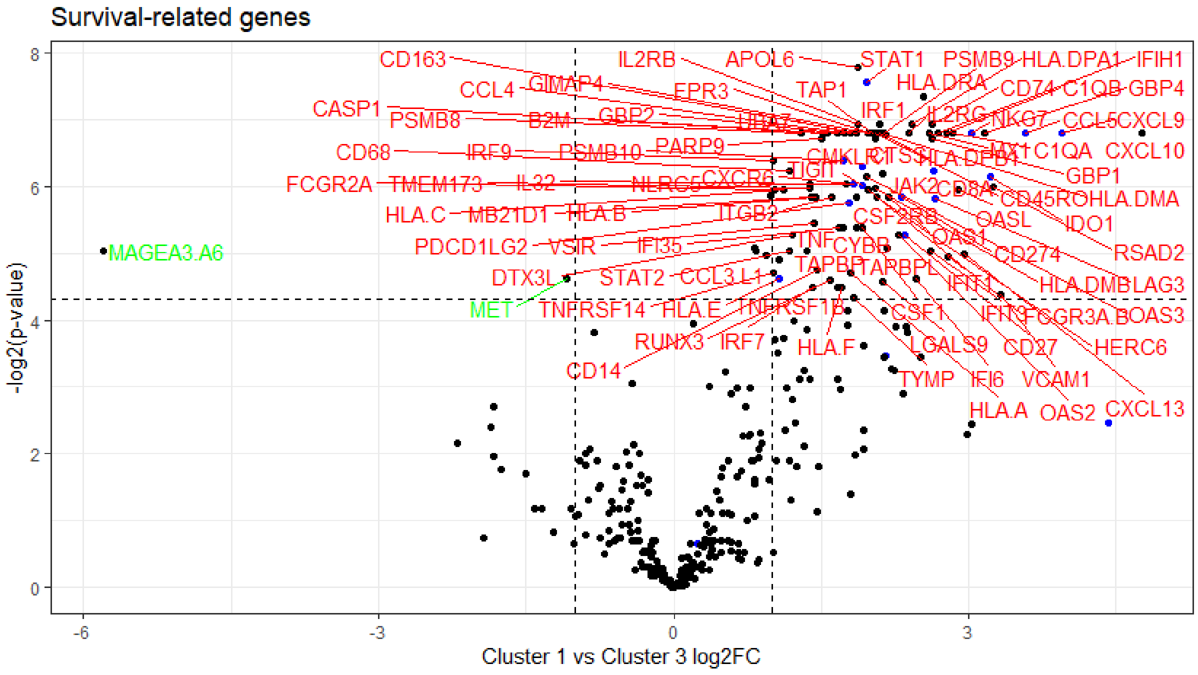

Supplementary Figure S3. Enrichment plots for significantly enriched signaling pathways. Genes are ranked and ordered based on log2-fold change between the conditions described in the header. NES=normalized enrichment score. (NED=No evidence of disease, DOD=dead of disease).

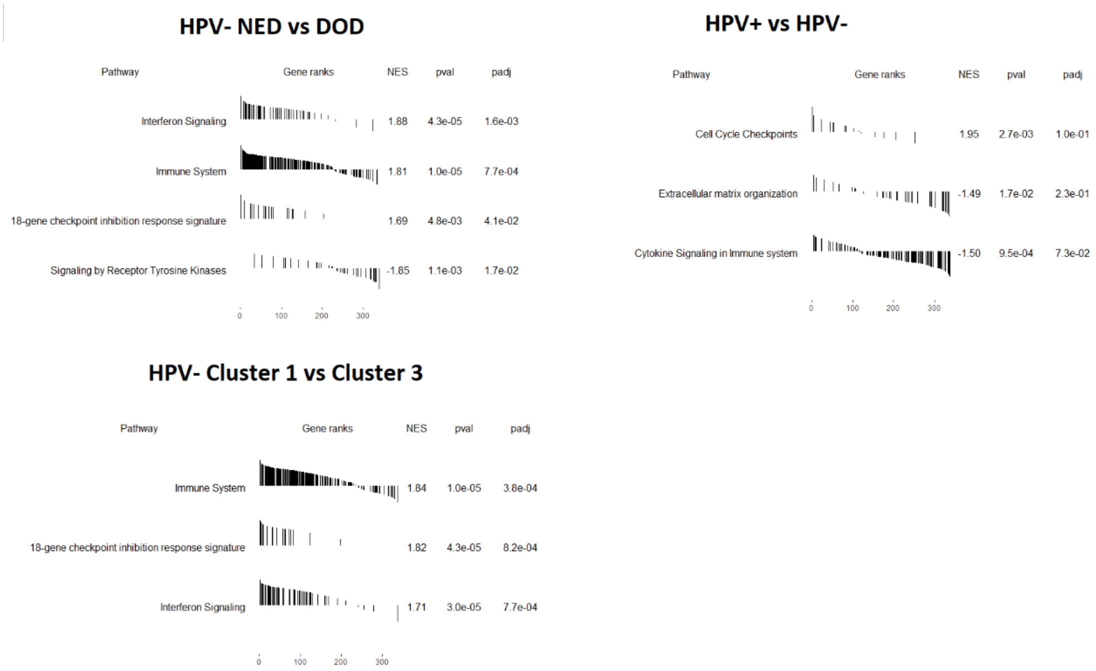

Supplementary Figure S4. Examples of HPSCC stained for S100A12 and HLA class I expression by IHC. (A-C) S100A12 staining showing; (A) absent expression, (B) medium expression and (C) strong expression. (D-F) HLA class I staining showing; (D) absent expression, (E) medium expression and (F) strong expression.

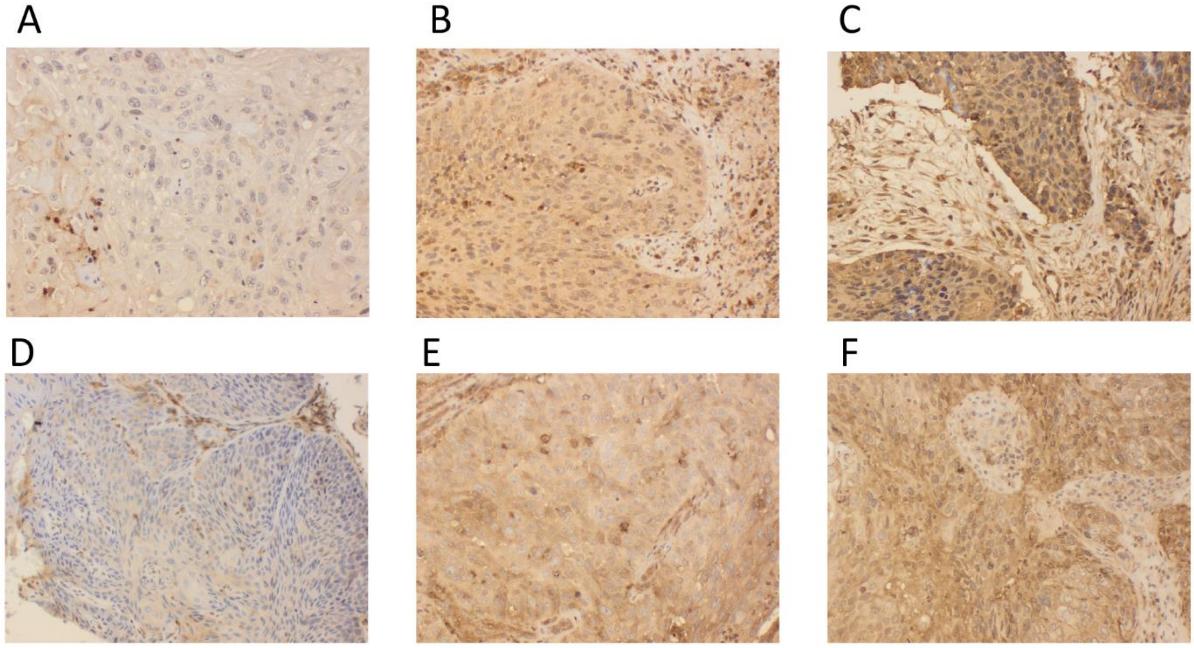

Supplementary Figure S5. Kaplan-Meier curves presenting progression free (A), disease free (B) and overall survival (C) for patients with HPV-negative HPSCC in relation to combined expression of HLA class I and numbers of CD8+ tumor infiltrating lymphocytes (TILs). HLA class I expression dichotomized between absent/low and medium/high. CD8+ TILs dichotomized HPSCC with the highest quartile of CD8+ cells and the three lowest quartiles combined. Both high (green), one high, one low (red) or both low (blue). Notches denotes censored.

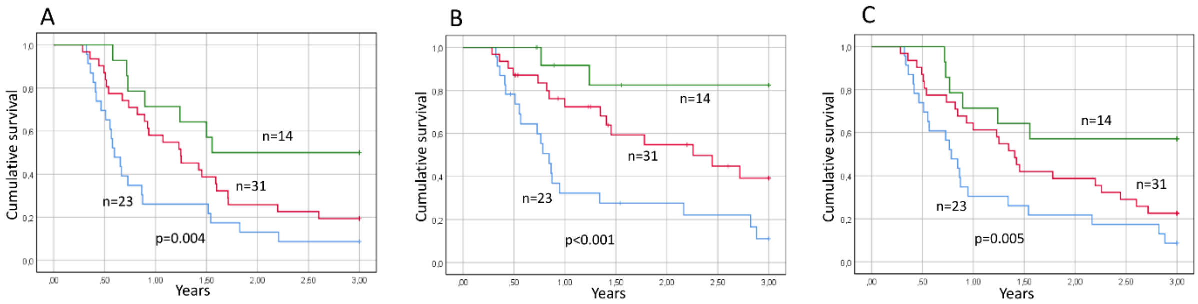

Supplementary Figure S6. Transcripts plotted by mean and variance. Mean and variance values are log2-transformed. Intercepting lines represent cutoffs set at the respective medians.

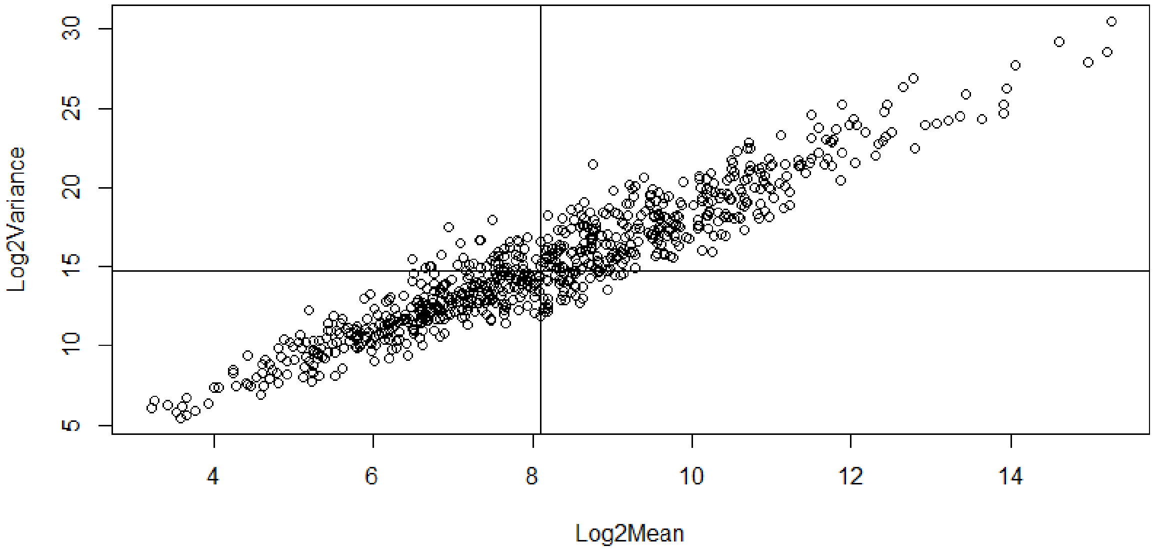

Supplement: Supplementary file 1 — Supplementary Figures. [file 41598_2020_80226_MOESM1_ESM.pdf]
